# Coach, sports medicine, and parent influence on concussion care seeking intentions and behaviors in collegiate student-athletes

**Published:** 2020-05-26

**Authors:** Julianne D. Schmidt, David Welch Suggs, Michelle L. Weber Rawlins, Laura Bierema, Lloyd Stephen Miller, Ron Courson, Fred Reifsteck

**Affiliations:** ^1^Department of Kinesiology, University of Georgia, Athens, Georgia, USA; ^2^UGA Concussion Research Laboratory, University of Georgia, Athens, Georgia, USA; ^3^Grady College of Journalism and Mass Communication, University of Georgia, Athens, Georgia, USA; ^4^Department of Interdisciplinary Health Sciences, AT Still University, Mesa, AZ, USA; ^5^Department of Lifelong Education, Administration, and Policy, University of Georgia, Athens, Georgia, USA; ^6^Department of Psychology, University of Georgia, Athens, Georgia, USA; ^7^Athletic Association, University of Georgia, Athens, Georgia, USA; ^8^University Health Center, University of Georgia, Athens, Georgia, USA

**Keywords:** concussion reporting, sport culture, concussion non-disclosure, mild traumatic brain injury, brain injury, socio-ecological model

## Abstract

**Background:**

Sport is a socio-ecological framework where student-athletes are part of a larger community of stakeholders, including coaches, sports medicine professionals (SMPs), and parents. This framework may hold influence over whether student-athletes seek care for a concussion.

**Aim:**

We aimed to describe, compare, and determine the influence of stakeholder concussion knowledge, attitudes, and concussion scenario responses.

**Materials and methods:**

We recruited a sample of 477 student-athletes and their 27 coaches (response rate=46.6%), 24 SMPs (48.7%), and 31 parents/guardians (4.8%). Stakeholder surveys assessed their concussion knowledge, attitudes toward care seeking, and concussion scenario responses. Surveys administered to student-athletes assessed their concussion care seeking intentions and behaviors. Kruskal–Wallis tests were used to compare responses between stakeholder groups and to determine the differences in student-athlete intentions and behaviors (alpha=0.05).

**Results:**

SMPs had significantly better knowledge (p<0.001) and concussion scenario responses (p<0.001) compared to both coaches and parents. SMPs also had significantly better attitudes compared to parents, but not coaches (p=0.038). Coach concussion scenario responses (p=0.044) and SMP knowledge positively influenced student-athletes’ concussion care seeking intentions (p=0.049). Parent responses were not associated with their child’s concussion care seeking intentions and behaviors.

**Conclusions:**

The gap in coach and parent concussion knowledge and concussion scenario response relative to SMPs is a preliminary target for stakeholder concussion education and supports the current sports medicine model where SMPs primarily disseminate concussion education. Stakeholders, specifically coaches and SMPs, do hold influence over collegiate athlete concussion care seeking intentions and behaviors.

**Relevance for patients:**

Stakeholders should be addressed within educational efforts aimed at student-athletes and should also complete stakeholder-specific concussion education.

## 1. Introduction

Concussion diagnosis is one of the most significant challenges currently facing the sports medicine community. Concussion diagnosis is often dependent on symptom reporting by the athlete. In 2004, McCrea *et al*. reported that 47.3% of all concussions go unreported [1]. In the 13 years since, researchers have replicated these results, finding concussion care seeking rates at ~50% [2-5]. Given the attention being paid to the injury, a rise in concussion care seeking rates and, therefore, a rise in concussion incidence should be expected. It is very important that athletes seek care for their concussion immediately to reduce their subsequent injury risk [6] and to reduce their risk of longer recovery [7].

The steady concussion care seeking rate potentially suggests that current knowledge-based concussion education efforts are not effective in improving concussion care seeking behaviors [8,9]. While a baseline level of knowledge is needed for student-athletes to recognize a concussion, student-athletes may still conceal a concussion because of intrinsic or extrinsic cultural pressure [10]. Sport is a socio-ecological framework and student-athletes are part of a larger community of stakeholders, such as coaches, parents, and sports medicine professionals (SMPs) [10]. However, the role that stakeholders play in influencing concussion care seeking decisions is not well understood.

Better understanding stakeholder influence might better inform concussion education design and implementation to be more effective at improving student-athletes concussion care seeking behaviors. Student-athletes who experienced pressure from coaches, parents, teammates, and fans are more likely to conceal a concussion and continue playing [11]. High school athletes, in particular, place great value on what others expect them to do [12]. It also seems that student-athletes are institutionalized to believe that their coach would not support their decision to seek care for a concussion [13].

SMPs likely also hold influence on student-athlete concussion care seeking behaviors. High school athletes with access to an athletic trainer had more concussion knowledge, but did not seek care for suspected concussions more frequently than athletes without access to an athletic trainer [14]. However, SMP influence may be greater in the collegiate sport setting, compared to high school, due to greater access to an athletic trainer, or team physician, which could possibly translate to an increased dissemination and transfer of concussion knowledge and beliefs. Further, collegiate athletes most commonly receive concussion education from their athletic trainer, but many indicate that they would also like coaches and physicians to be involved [15].

Parents are another important stakeholder group that likely hold influence [16]. However, collegiate student-athletes typically move away from their childhood home and away from the direct influence of their parents at the time they begin playing collegiate sports, which may modify the influence of parents in the collegiate environment.

Concussion educational requirements have been mandated by state laws and sport organizations, but little is known about how these educational efforts can be targeted. Therefore, the primary purpose of this study was to describe and compare concussion knowledge, attitudes, and concussion scenario responses between stakeholder groups. Although it seems logical that stakeholders influence student-athletes’ concussion care seeking, no previous studies have directly linked the knowledge, attitudes, and concussion scenario responses of stakeholders with the concussion care seeking intentions and behaviors of student-athletes. Thus, our secondary purpose was to examine the influence of stakeholder knowledge, attitudes, and concussion scenario responses on student-athlete concussion care seeking intentions and behaviors.

## 2. Materials and Methods

### 2.1. Sampling and survey administration

We recruited from a total sample of 1140 student-athletes and their 58 coaches, 640 parents/guardians, and 39 SMPs at a single Division I university. Surveys were sent to all stakeholders through an email imbedded Qualtrics (Qualtrics LLC, Provo, UT) link between August 2016 and April 2017. Follow-up emails were then sent weekly for 4 weeks. Coaches and SMPs that did not respond were given a paper survey [17]. Stakeholder surveys assessed knowledge, attitudes, and concussion scenario responses. Student-athletes completed surveys regarding concussion care seeking intentions and behaviors. Survey section and question order were randomized where appropriate. All surveys contained an operational definition of concussion and are detailed in Appendix 1 [12]. All participants completed an institutional review board approved consent form.

### 2.2. Stakeholder survey

Participants responded on a 7-point scale, with responses ranging from “strongly disagree” (=1), “disagree” (=2), “somewhat disagree” (=3), “neutral” (=4), “somewhat agree” (=5), “agree” (=6), to “strongly agree” (=7) for all survey components (Appendix 1). A pilot administration of the survey was conducted with parents/guardians (n=24) and SMPs (n=14). The survey had fair to excellent item level internal consistency for knowledge (α=0.70-0.79), attitudes (α=0.55-0.63), and concussion scenario response (α=0.79-0.81). Survey tools were developed by Rosenbuam [18] and Register-Mihalik [12], but later adapted by Kroshus [8]

#### 2.2.1. Stakeholder knowledge

Stakeholders were asked to respond to thirteen statements regarding concussion. Participants responded to statements such as “People who have had a concussion are more likely to have another concussion” and “A concussion may cause an athlete to feel depressed or sad.” Responses to two statements (“The brain never fully heals after a concussion” and “Concussions pose a risk to an athlete’s long-term health and well-being”) were excluded from analysis because the science is unsettled in these areas and determining which direction indicated a correct response was not possible (11 total questions remained).

#### 2.2.2. Stakeholder attitudes

Participants responded to eight statements regarding outcomes of concussion care seeking, such as “If an athlete reports what they suspect might be a concussion, they will hurt their team’s performance” and “The sooner an athlete reports a concussion the sooner they will be back at full strength.”

#### 2.2.3. Stakeholder concussion scenario responses

Stakeholders read and responded to questions regarding four scenarios. We adapted items to reference three stakeholder perspectives. Example responses are presented in Table 1.

**Table 1:** Example concussion scenario and question formats for each stakeholder group. “Scenario – Athlete M experienced a concussion during the first game of the season. Athlete O experienced a concussion of the same severity during the semifinal playoff game. Both athletes had persisting symptoms.”

	Question 1	Question 2	Question 3
Coaches	Athletes on my team…	Most athletes…	Most coaches…
Parents/Guardians	Athletes on my child’s team …	Most athletes…	Most parents…
Sports medicine professionals	Athletes at my university…	Most athletes…	Most sports medicine professionals …
	…would feel that Athlete M should have returned to play during the first game of the season.	…would feel that Athlete M should have returned to playing during the first game of the season.	…would feel that Athlete M should have returned to playing during the first game of the season.

### 2.3. Student-athlete survey

Student-athlete surveys captured two forms of intentions and behaviors: Symptom care seeking and concussion care seeking; for a total of four outcome measures (symptom care seeking intentions, concussion care seeking intentions, symptom care seeking behaviors, and concussion care seeking behaviors) (Appendix 1). Both forms were assessed to account for differences in how student-athletes might personally define a concussive event. In surveys related to symptom care seeking, questions were oriented toward determining intentions and behaviors on the occurrence of specific concussion symptoms, whereas concussion care seeking surveys more directly assessed student-athletes’ global intentions and behaviors on the occurrence of a concussive event. A pilot survey administration given to student-athletes (n=64) revealed fair to excellent item level internal consistency and total score test-re-test reliability for intentions (α=0.92, ICC_2,1_: 0.52) and behaviors (α=0.88, ICC_2,1_: 0.45) [19].

#### 2.3.1. Student-athlete care seeking intentions

Symptom care seeking intentions were assessed by asking student-athletes to review a list of eight common symptoms of concussions and rate whether they intended to immediately report the presence of each symptom to their coach or athletic trainer if the symptoms were experienced after an impact [8,20]. Concussion care seeking intentions were measured by asking student-athletes to rate whether they intended, planned, and would make an effort to report a possible concussion [12].

#### 2.3.2. Student-athlete care seeking behaviors

Symptom care seeking behaviors were assessed by asking student-athletes whether during the previous 365 days they had experienced any of eight listed symptoms after sustaining an impact and then whether they immediately told a coach or athletic trainer [8,20]. Concussion care seeking behaviors were captured by asking student-athletes to report the number of concussions and the number of “bell ringer/ding” episodes sustained and then how many of those were reported to a medical professional (doctor, athletic trainer, etc.) or a coach [2].

### 2.4. Data and statistical analysis

Responses were reverse-coded where appropriate and averaged across items to create a composite score. Higher scores indicate better responses for stakeholder: Knowledge, attitudes, and concussion scenario responses; and for student-athlete: Symptom care seeking intentions and concussion care seeking intentions. Symptom care seeking behavior item responses were used to dichotomize student-athletes as “care seekers” and “non-care seekers,” excluding those that did not experience any concussion symptoms. Concussion care seeking behaviors were expressed as a percentage ([number of concussions+dings and bell ringers reported]/[number of concussions+dings and bell ringers sustained] *100), excluding those that did not sustain a concussion or ding/bell ringer. Table 2 shows the distribution of stakeholders across sports.

**Table 2:** Demographic information for stakeholders and student-athletes.

Sport	Coach (n=27) n (%)	Sports medicine (n=24) n (%)	Parents (n=31) n (%)	Student-athletes (n=297) n (%)
Sex				
Female	8 (29.6)	14 (58.3)	NA	173 (58.2)
Male	19 (70.4)	10 (41.7)	NA	124 (41.8)
Working with men’s sports				
Baseball	3 (11.1)	1 (4.2)	1 (3.2)	30 (10.1)
Basketball	1 (3.7)	1 (4.2)	0 (0.0)	6 (2.0)
Football	1 (3.7)	4 (8.3)	1 (3.2)	0 (0.0)
Golf	1*(3.7)	1* (4.2)	1 (3.2)	4 (1.3)
Swimming/Diving	0* (0.0)	2* (8.3)	2 (6.5)	3 (1.0)
Tennis	2 (7.4)	2 (8.3)	1 (3.2)	47 (15.8)
Track/Cross country	5* (18.5)	1* (4.2)	1 (3.2)	13 (4.4)
Working with women’s sports				
Basketball	4 (14.8)	2 (8.3)	1 (3.2)	12 (4.0)
Equestrian	2 (7.4)	1 (4.2)	7 (22.6)	45 (15.2)
Golf	2*(7.4)	1* (4.2)	0 (0.0)	5 (1.7)
Gymnastics	1 (3.7)	0 (0.0)	0 (0.0)	11 (3.7)
Soccer	2 (7.4)	1 (4.2)	2 (6.5)	6 (2.0)
Softball	2 (7.4)	0 (0.0)	2 (6.5)	20 (6.7)
Swimming/Diving	0* (0.0)	2* (8.3)	6 (19.4)	13 (4.4)
Tennis	2 (7.4)	1 (4.2)	0 (0.0)	16 (5.4)
Track/Cross country	5* (18.5)	1* (4.2)	2 (6.5)	34 (11.4)
Volleyball	0 (0.0)	2 (8.3)	4 (12.9)	11 (3.7)
No single sport association	0 (0.0)	5 (20.8)	0 (0.0)	0 (0.0)
Years of eligibility				
1 year remaining	NA	NA	NA	76 (25.6)
2 years remaining	NA	NA	NA	62 (20.9)
3 years remaining	NA	NA	NA	85(28.6)
4 years remaining	NA	NA	NA	74 (24.9)
Age (years)	NA	NA	NA	19.7±1.4

* Coaching and sports medicine staff are the same for both men’s and women’s teams. NA: Not applicable (not captured)

All data were analyzed using SPSS 24.0 with an a priori alpha level of 0.05. None of the outcome variables were normally distributed. To address our primary purpose, we used Kruskal–Wallis tests to compare: Knowledge, attitudes, and concussion scenario responses (questions 1, 2, and 3 separately) between stakeholder groups (coaches, SMPs, and parents). In cases of significance, a Mann–Whitney U *post hoc* test was used.

Data for coaches and SMPs were not independent observations because several athletes associate with each individual. Accordingly, we created stakeholder median groupings by determining the median response across all respondents on the team level. For example, if two coaches from one team responded and together had a median response of six (agree) on the knowledge survey section, we assigned all athletes on that team to a coach knowledge agree median grouping. We then compared student-athlete symptom care seeking intentions, concussion care seeking intentions, and concussion care seeking behaviors between the median groupings for knowledge, attitudes, and concussion scenario responses (question 1 only – my athletes) using Kruskal–Wallis tests. In cases of significance, a Mann–Whitney U *post hoc* test was used. We excluded massage therapists, sport psychologists, and team physicians for analyses regarding our secondary purpose because these individuals associated with all or most teams and could not be directly linked to any athlete subsets by team. We conducted a Chi-square analysis (Fisher’s exact if cell counts <5) to determine the association between the stakeholder response and athlete behavior (care seeker, and non-care seeker). Finally, we analyzed the team level (team average) association between concussion care seeking (symptom care seeking intentions, concussion care seeking intentions, and concussion care seeking behaviors) and stakeholder responses (coach/SMP knowledge, attitudes, and concussion scenario responses – question 1 only – my athletes) using Spearman’s rho. Parent data were matched individually with their child’s data. We analyzed the association between parent knowledge, attitudes, and concussion scenario responses (question 1 only – my athletes) with their child’s symptom care seeking intentions, concussion care seeking intentions, and concussion care seeking behaviors using Spearman’s rho. We chose to use concussion scenario response question 1 only because questions 2-3 were worded more broadly beyond the student-athletes the stakeholders directly interacted with.

## 3. Results

### 3.1. Stakeholder group comparisons

**Figure 1:**
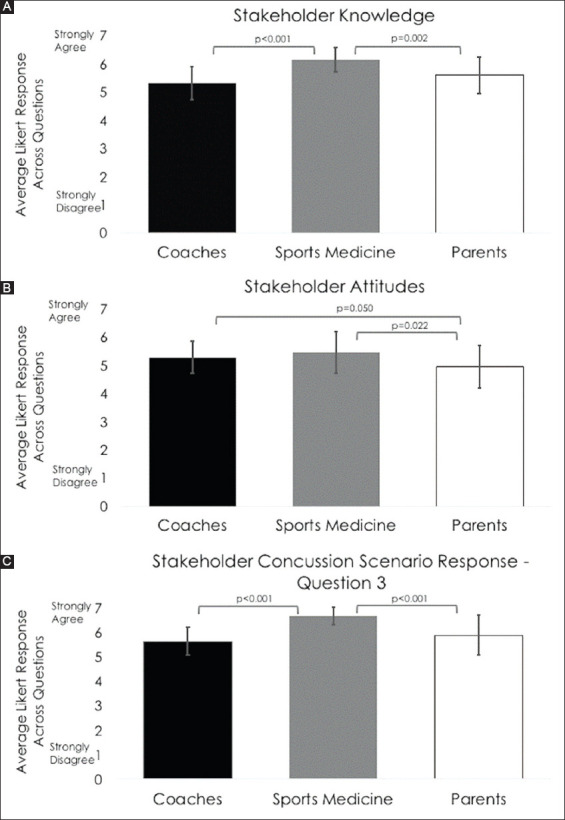
(**A-C**) Stakeholder comparisons of knowledge, attitudes, and concussion scenario responses.

Stakeholder group descriptives for each outcome variable are detailed in Table 3. Stakeholder groups differed on knowledge (χ^2^(2)=22.4, *P*<0.001), attitudes (χ^2^(2)=6.5, *P*=0.038), and for concussion scenario responses – question 3 (most stakeholders – Table 1, e.g., questions) (χ^2^(2)=29.8, *P*<0.001). For knowledge, SMPs (6.16±0.43) had significantly higher knowledge compared to both coaches (5.33±0.58, *P*<0.001) and parents (5.61±0.65, *P*=0.002) (Figure 1a). Coaches and parents had similar knowledge (*P*=0.081). For attitudes, SMPs (5.45±0.74) had significantly better attitudes compared to parents (4.95±0.75, *P*=0.022), but did not differ from coaches (5.28±0.57, *P*=0.466) (Figure 1b). Coaches had marginally better attitudes compared to parents (*P*=0.050). For concussion scenario responses – question 3 (most stakeholders), SMPs more strongly agreed that “most SMPs” would have more favorable scenario responses (6.68±0.36) compared to how coaches responded for how “most coaches” would react to the injury scenarios (5.65±0.58, *P*<0.001) and compared to how parents responded for how “most parents” would react to the injury scenarios (5.89±0.82, *P*<0.001) (Figure 1c). Coaches and parents responded similarly (*P*=0.121). There were no differences between stakeholder groups for concussion scenario responses – question 1 (my athletes) (*P*=0.762) or question 2 (most athletes) (*P*=0.644). Mann–Whitney U *post hoc* test results for between groups differences are presented in Figure 1.

**Table 3:** Descriptive results for stakeholder groups and student-athletes.

	Mean±SD	Median	Nearest response category to median	95% CI
Coaches (n=27)				
Knowledge^1^	5.33±0.58	5.45	Somewhat agree	5.10, 5.55
Attitudes^2^	5.28±0.57	5.38	Somewhat agree	5.06, 5.51
Concussion scenario responses				
Q1: Athletes on my team	5.58±0.72	5.67	Agree	5.30, 5.86
Q2: Most athletes	5.33±0.71	5.33	Somewhat agree	5.05, 5.61
Q3: Most coaches^3^	5.65±0.58	5.50	Agree	5.42, 5.88
SMPs (n=24)				
Knowledge^1^	6.16±0.43	6.23	Agree	5.97, 6.34
Attitudes^2^	5.45±0.74	5.38	Somewhat agree	5.15, 5.77
Concussion				
Q1: Athletes at my university	5.55±1.00	5.83	Agree	5.13, 5.97
Q2: Most athletes	5.10±0.89	5.17	Somewhat agree	4.72, 5.47
Q3: Most SMPs^3^	6.68±0.36	6.83	Strongly agree	6.65, 6.84
Parents (n=31)				
Knowledge^1^	5.61±0.65	5.55	Agree	5.37, 5.86
Attitudes^2^	4.95±0.75	5.19	Somewhat agree	4.68, 5.23
Concussion scenario responses:				
Q1: Athletes on my child’s team	5.44±0.93	5.50	Somewhat agree	5.10, 5.79
Q2: Most athletes	5.33±1.07	5.67	Agree	4.93, 5.73
Q3: Most parents	5.89±0.82	6.08	Agree	5.58, 6.20

*SMPs: Sports medicine professionals.

1 SMPs >coaches (P<0.001) and parents (*P*=0.002). Coaches and parents had similar knowledge (*P* =0.081) - (χ^2^(2)=22.4, *P*<0.001).

2 SMPs >parents (*P*=0.022), but did not differ from coaches (*P*=0.466). Coaches marginally >parents (*P*=0.050) - (χ^2^(2)=6.5, *P*=0.038).

3 SMPs >coaches (*P*<0.001) and parents (*P*<0.001). Coaches=parents (*P*=0.121) - (χ^2^(2)=29.8, *P*<0.001)

### 3.2. Coaches influence

A total of 477 student-athletes completed the intentions and behaviors surveys, 297 of which matched to at least one stakeholder. Twenty-seven coaches from 14 teams completed the survey (response rate=46.6%). Median response values ranged from 5 to 6 for knowledge, 5 to 7 for attitudes, and 6 to 7 for concussion scenario responses. Student-athlete intentions and behaviors were not significantly influenced by coach knowledge (*P*=0.084-0.658) or attitudes (*P*=0.063-0.182). However, student-athletes on teams with coaches that strongly agreed with concussion scenario response – question 1 (my athletes) (n=16, 6.4±1.1) had significantly better concussion care seeking intentions compared to those with coaches that agreed (n=241, 5.9±1.2; χ^2^(1)=4.1, *P*=0.044). Symptom care seeking behaviors were not associated with coach median responses for any outcome variable (*P*=0.209-0.704). Team level correlations between student-athlete intentions and behaviors and coach knowledge (*P*=0.132-0.291), attitudes (*P*=0.537-0.932), and concussion scenario responses (*P*=0.325-0.488) were not significant.

### 3.3. SMP influence

Nineteen athletic trainers and five other sports medicine staff members from 15 teams completed the survey (response rate=48.7%). Median response values ranged from 6 to 7 for knowledge, 4 to 7 for attitudes, and 4 to 7 for concussion scenario responses. Student-athletes with SMPs that strongly agreed (n=16, 61.4±47.0%) with knowledge survey questions, sought care for a significantly higher percentage of their concussions (concussion care seeking behavior) compared to those with SMPs that agreed (n=41, 34.3±46.7%; χ^2^(1)=3.88, *P*=0.049). Student-athlete intentions and behaviors did not differ across SMP attitude groupings (*P*=0.109-0.926). Concussion care seeking intentions significantly differed across SMP concussion scenario response – question 1 groupings (χ^2^(1)=13.3, *P*=0.004). Concussion care seeking intentions were significantly higher among student-athletes that had SMPs that were neutral (n=51, 6.4±0.9) compared to those that had SMPs that agreed (n=90, 5.8±1.2, *P*=0.001) and strongly agreed (n=45, 5.9±1.2, *P*=0.013). Student-athletes with SMPs that somewhat agreed (n=87, 6.0±1.3) had significantly higher concussion care seeking intentions compared to those that agreed (n=90, 5.8±1.2, *P*=0.027). Symptom care seeking behaviors were not associated with SMP median responses for any outcome variable (*P*=0.321-0.781). Team level correlations between student-athlete intentions and behaviors and SMP knowledge (*P*=0.190-0.352), attitudes (*P*=0.229-0.888), and concussion scenario responses (*P*=0.167-0.966) were not significant.

### 3.4. Parents

Thirty-one parents completed the survey (response rate=4.8%). Median response values ranged from 4 to 7 for knowledge, 2 to 7 for attitudes, and 4 to 7 for concussion scenario responses. Parent knowledge, attitudes, and concussion scenario responses were not associated with their child’s symptom care seeking intentions (*P*=0.083-0.752), concussion care seeking intentions (*P*=0.414-0.911), or concussion care seeking behaviors (*P*=0.102-0.896).

## 4. Discussion

The results of this study highlight differences in concussion knowledge, attitudes, and concussion scenario response across stakeholder groups. As is expected given their extensive medical training and clinical experience, SMPs had better concussion knowledge and concussion scenario response compared to coaches and parents. We also revealed that coach concussion scenario response and SMP knowledge influenced student-athlete concussion care seeking intentions. These variables may be key targets for improving the culture of concussion care seeking among collegiate student-athletes.

### 4.1. Stakeholder group comparisons

SMPs had better concussion knowledge and concussion scenario response compared to coaches and parents. The group consisted of mostly athletic trainers and five other SMPs (sport psychologist, team physician, massage therapist, sport nutrition, etc.). The gap in concussion knowledge and concussion scenario response between SMPs and coaches/parents might serve as a preliminary target for stakeholder concussion education and supports the current sports medicine model where SMPs have taken primary responsibility of disseminating concussion education [15]. However, this does not mean that SMPs should solely bear this responsibility, as the involvement of other stakeholder groups may be valuable to student-athletes [15]. For example, a SMP might organize and determine the content of a concussion education session, but might invite members of the coaching staff to convey certain information. Messages such as “you should seek care for a suspected concussion,” might be more meaningful or memorable if coming from a coach rather than a SMP.

Interestingly, SMPs and coaches had similar attitudes. Attitude survey questions all began with “If an athlete reports what they suspect might be a concussion,…” and then listed eight potential outcomes (e.g. “they will lose their spot in the line-up”). It seems possible that SMPs would see these potential outcomes as inevitable, despite the knowledge they have and the high value they place on prioritizing health. Although both coaches and SMPs may sympathize with a concussed athlete and want them to seek care their concussion [21], there may be little that either group can do prevent the negative outcomes that come with missed playing time following injury. Parents, however, had a slightly more pessimistic view of the potential outcomes that might follow if an athlete was to sustain a concussion.

### 4.2. Stakeholder group influence on concussion care seeking

Overall, the coaches in our sample either agreed or strongly agreed with favorable responses to the scenarios regarding their athletes (question 1), but even the step off between strongly agree and agree resulted in significantly lower concussion care seeking intentions among the student-athletes on those teams. It is possible that student-athletes sense the extent to which their coach values concussion care seeking by their actions and adapt their intentions accordingly. Learning a behavior can occur from direct instruction, through observation, and/or through experience [22]. If a coach does not explicitly endorse concussion care seeking, the student-athletes may rely on their observation of behavior and/or their experiences. Our results regarding the concussion scenario responses may mimic the coaching behaviors that student-athletes have observed or experienced and may ultimately have influenced their concussion care seeking intentions. Another proposed approach that warrants consideration in the context of stakeholder influence is the Social Norms Theory. Student-athletes misperceive their teammates concussion care seeking norms [19] and it is possible that they may also misperceive the concussion care seeking norms of other stakeholder groups. Thus, stakeholder involvement in concussion education might advance efforts by correcting misperceived norms. For example, student-athletes present at a concussion education session where their coach is absent might misperceive the absences as a lack of support for the information. The presence, positive endorsement, and involvement of the coach might advance the messaging by correcting this misperception.

The SMPs were the most knowledgeable stakeholder groups. The SMP group had little variability in their responses, but even the slight difference between SMPs that strongly agreed and agreed influenced student-athlete concussion care seeking behaviors. SMPs that are more knowledgeable about concussion may convey that knowledge to student-athletes on their teams. That increase in knowledge could potentially cause student-athletes to be better at recognizing the initial injury and understanding the importance of seeking care for the injury because of its seriousness. High school student-athletes with access to an athletic trainer have better concussion knowledge [14,23] underscoring the important role SMPs plays in concussion culture. We found that student-athlete concussion care seeking intentions were actually better if their SMPs had more neutral concussion scenarios responses. It is not immediately evident why this might be, but it is possible that there is an inverse relationship between the optimistic projections SMPs make about how most athletes at their university would respond to various concussion scenarios and the actual strength of the athletes’ intentions.

Arguably, the most interesting finding from our study was the difficulty engaging parent participation and the subsequent low response rate. Parent responses were not associated with student-athlete concussion care seeking intentions and behaviors. Null findings should be interpreted with caution, but provide preliminary evidence regarding parent influence at the collegiate level. Kroshus *et al*. [16] found that parents that perceived concussion as a threat were more likely to encourage their child to seek care for a concussion. However, parents that had higher investments in the child’s sport achievement were less likely to encourage care seeking. At the Division I collegiate level, it is very possible that parents hold high investment in their child’s sport achievement, however, we cannot determine the extent to which this may have influenced our results. Our sample of parents came predominantly from non-contact sports and may have lacked previous experience with concussion [16].

Our study was limited. As is true with most survey research, self-reported survey responses are prone to the social desirability and recall biases. Further, our study was cross-sectional and our sample came from a single Division I university. Future research is needed to determine how stakeholder influence differs across other levels of play. Although we provide preliminary evidence regarding parent influence, our sample and response rate were small. Parents and guardians can be difficult to contact and engage at the collegiate level, given their geographic distance and separation from the program. More research is needed in this group and other parent groups.

## 5. Conclusion

Our findings support the current sports medicine model where SMPs are primarily responsible for disseminating concussion education, but also highlight the potential value of involvement of coaches. Stakeholders, specifically coaches and SMPs, do hold influence over student-athlete concussion care seeking. Further, the influence of stakeholders on student-athletes extended beyond just concussion knowledge. Thus, stakeholder concussion education should address attitudes and concussion scenario responses, in addition to concussion knowledge. We recommend not only that stakeholders be addressed in educational efforts aimed at student-athletes but also that stakeholders complete stakeholder-specific concussion education as is currently required by some state concussion laws and sport organizations.

## Appendix

**Appendix 1 – Survey Tools**

**STAKEHOLDER SURVEY**

**Knowledge**

Directions: These statements about concussions may or may not be true. Please rate how strongly you agree with each statement.

Response Options: Strongly Disagree, Disagree, Somewhat Disagree, Neither Agree nor Disagree, Somewhat Agree, Agree, Strongly Agree

People who have had a concussion are more likely to have another concussion.

There is a possible risk of death if a second concussion occurs before the first one has healed.

A concussion cannot cause brain damage unless the person has been knocked out.

It is easy to tell if a person has a concussion by the way the person looks or acts.

Symptoms of a concussion can last for several weeks.

Resting your brain by avoiding things such as playing video games, texting, and doing schoolwork is important for concussion recovery.

After a concussion occurs, brain imaging (e.g., computer assisted tomography scan, magnetic resonance imaging, Xray, etc.) typically shows visible physical damage to the brain (e.g., bruise, blood clot).

A concussion may cause an athlete to feel depressed or sad.

Once an athlete feels “back to normal,” the recovery process is complete.

Even if a player is experiencing the effects of a concussion, performance on the field of play will be the same as it would be had the player not experienced a concussion.

A concussion can only occur if there is a direct hit to the head.

**Attitudes**

Directions: Please rate how strongly you agree with each statement.

Response Options: Strongly Disagree, Disagree, Somewhat Disagree, Neither Agree nor Disagree, Somewhat Agree, Agree, Strongly Agree

If an athlete reports what they suspect might be a concussion, they will hurt they’re team’s performance.

If an athlete reports what they suspect might be a concussion, they will not be allowed to start playing or practicing when they think they’re ready.

If an athlete reports what they suspect might be a concussion, they will lose their spot in the line-up.

If an athlete reports what they suspect might be a concussion, they’re teammates will think less of them.

The sooner an athlete reports a concussion the sooner they’ll be back at full strength.

If an athlete reports what they suspect might be a concussion, they will be held out of upcoming games even if it is NOT a concussion.

If an athlete reports what they suspect might be a concussion, they’re teammates will think they made the right decision.

If an athlete reports what they suspect might be a concussion, they will be better off in the long run.

**Concussion Scenario Response (Coach Version Shown)**

Directions: Please read each of the following scenarios and rate how strongly you agree or disagree with the statements that follow.

Response Options: Strongly Disagree, Disagree, Somewhat Disagree, Neither Agree nor Disagree, Somewhat Agree, Agree, Strongly Agree

*Scenario 1: Athlete M experienced a concussion during the first game of the season. Athlete O experienced a concussion of the same severity during the semifinal playoff game. Both athletes had persisting symptom*s.

Athletes on my team would feel that Athlete M should have returned to play during the first game of the season.

Most athletes would feel that Athlete M should have returned to playing during the first game of the season.

Most coaches would feel that Athlete M should have returned to playing during the first game of the season.

Athletes on my team would feel that Athlete O should have returned to play during the semifinal playoff game.

Most athletes would feel that Athlete O should have returned to playing during the semifinal playoff game.

Most coaches would feel that Athlete O should have returned to playing during the semifinal playoff game.

*Scenario 2: Player R experiences a concussion during a game. Coach A decides to keep Player R out of the game. Player R’s team loses the game*.

Athletes on my team would feel that Coach A made the right decision to keep Player R out of the game.

Most athletes would feel that Coach A made the right decision to keep Player R out of the game.

Most coaches would feel that Coach A made the right decision to keep Player R out of the game.

*Scenario 3: Athlete R experiences a concussion. Athlete R’s team has an athletic trainer on the staff*.

Athletes on my team would feel that the athletic trainer, rather than Athlete R, should make the decision about returning Athlete R to play.

Most athletes would feel that the athletic trainer, rather than Athlete R, should make the decision about returning Athlete R to play.

Most coaches would feel that the athletic trainer, rather than Athlete R, should make the decision about returning Athlete R to play.

*Scenario 4: Athlete H experienced a concussion and has a game later in the day. He is still experiencing symptoms of concussion. However, Athlete H knows that if he tells his coach about the symptoms, his coach will keep him out of the game*.

Athletes on my team would feel that Athlete H should tell his coach about the symptoms.

Most athletes would feel that Athlete H should tell his coach about the symptoms.

Most coaches would feel that Athlete H should tell his coach about the symptoms.

Athletes on my team would continue playing while also having a headache that resulted from a minor concussion.

Most athletes would continue playing while also having a headache that resulted from a minor concussion.

Most coaches would continue playing while also having a headache that resulted from a minor concussion.

**STUDENT-ATHLETE SURVEY**

**Symptom Reporting Intentions**

Directions: Please rate how strongly you agree with the following statement: “I would stop playing and report my symptoms if I sustained an impact that caused me to.” Response Options: Strongly Disagree, Disagree, Somewhat Disagree, Neither Agree nor Disagree, Somewhat Agree, Agree, Strongly Agree

See stars

Vomit or feel nauseous

Have a hard time remembering things

Have problems concentrating on the task at hand

Feel sensitive to light or noise

Have a headache Experience dizziness or balance problems

Feel sleepy or in a fog

**Concussion Reporting Intentions**

Directions: Rate on how strongly you agree with the following statement: “When I experience possible concussion symptoms…”

Response Options: Strongly Disagree, Disagree, Somewhat Disagree, Neither Agree nor Disagree, Somewhat Agree, Agree, Strongly Agree

I intend to report

I plan to report

I will make an effort to report

**Symptom Reporting Behaviors**

Directions: Please read the following statements. Please check YES if the following has occurred to you in the past calendar year (i.e., the past 365 days) and click NO if it has not occurred to you in the past calendar year:

Response Options: Yes, No

Dizziness after an impact

Had my bell rung

Lost consciousness or blacked out after an impact

Saw stars after an impact

Vomited or felt nauseous after an impact

Forgot what to do while playing my sport after an impact

Had a headache at least once during the week after an impact

Had problems studying, concentrating or doing class work after an impact

----If all above = NO → excluded

Experienced any of these symptoms after an impact but did not immediately tell a coach or athletic trainer (e.g., kept playing in a practice or game) [categorized as a non-reporter if YES and reporter if NO]

Continued to experience any of these symptoms the day after a hit but did not tell a coach or athletic trainer [categorized as a non-reporter if YES and reporter if NO]

**Concussion Reporting Behavior**

In the past 365 days, how many possible concussions do you think you have experienced? Response Options: Drop down 1-10+

For the first (…) concussion you sustained in the past 365 days, did you report this concussion to a medical professional (doctor, athletic trainer, etc) or a coach? Response Options: Yes, No

In the past 365 days, how many possible dings/bell-ringers do you think you have experienced? Response Options: Drop down 1-10+

For the first (…) ding/bell-ringer you sustained in the past 365 days, did you report this episode to a medical professional (doctor, athletic trainer, etc) or a coach? Response Options: Yes, No
